# High-purity X-ray polarimetry at high energies for Mössbauer spectroscopy

**DOI:** 10.1107/S1600577526008702

**Published:** 2026-09-14

**Authors:** Berit Marx-Glowna, Ingo Uschmann, Robert Loetzsch, Willi Hippler, Judith Görs, Tony Wunderlich, Sakshath Sadashivaiah, Martin Tolkiehn, Gerhard G. Paulus, Ralf Röhlsberger

**Affiliations:** a Helmholtz Centre for Heavy Ion Research (GSI), Planckstr. 1, D-64291Darmstadt, Germany; b Helmholtz Institute Jena Fröbelstieg 3 D-07743Jena Germany; c Institute of Optics and Quantum Electronics Friedrich Schiller University Jena Max-Wien-Platz 1 D-07743Jena Germany; d DESY Photon Science, Notkestr. 85, D-22607Hamburg, Germany; RIKEN SPring-8 Center, Japan

**Keywords:** high-purity X-ray polarimetry, synchrotron Mössbauer spectroscopy, Mössbauer isotopes^151^Eu,^149^Sm,^161^Dy,^40^K, X-ray diffraction at high energies, X-ray multiple wave diffraction at high energies

## Abstract

High-purity X-ray polarimetry has so far been established only in the low-energy range up to 14.4 keV and has therefore been applied exclusively to the Mössbauer isotope ^57^Fe. Here we extend high-purity X-ray polarimetry to photon energies between 15 and 30 keV. Using asymmetric channel-cut Si crystals operated close to the Brewster angle, we design and realize a polarimeter optimized for synchrotron-based Mössbauer spectroscopy in this range of transition energies. As a demonstration, we implement a four-bounce Si (5 7 11) polarimeter at a photon energy of 22.494 keV at PETRA III and achieve a polarization purity of (1.2 ± 0.5) × 10^−8^. This establishes the polarimeter performance required for synchrotron Mössbauer spectroscopy at this energy and shows, supported by dynamical-diffraction calculations, that the method can be extended to nuclear transitions up to about 30 keV—opening access to isotopes such as ^151^Eu, ^149^Sm, ^119^Sn, and ^161^Dy. A nuclear-resonant measurement on these isotopes is the natural next step at a dedicated high-brilliance beamline.

## Abstract 2

This work extends high-purity X-ray polarimetry into the 15–30 keV range, enabling polarization-filtered synchrotron Mössbauer spectroscopy of higher-energy nuclear resonances. A proof-of-principle experiment at 22.494 keV demonstrates a polarization purity of (1.2 ± 0.5) × 10^−8^, establishing the feasibility of the approach.

## 1. Introduction

With the discovery of recoilless nuclear resonance absorption by Rudolf Mössbauer in 1958 (Mössbauer, 1958 ▸), Mössbauer spectroscopy became an important research technique in all natural sciences, mostly in solid state physics, materials science, biophysics and chemistry. Recoilless nuclear resonance absorption/emission makes it possible to use the natural line width of nuclear transitions for spectroscopy, which typically lie in the range 10^−7^ to 10^−11^ eV for most Mössbauer isotopes. This enables high-precision measurements of hyperfine interactions and provides detailed information on charge densities as well as internal electric and magnetic fields in condensed matter.

Modern X-ray sources make it possible to excite nuclear levels from the ground state to perform classical Mössbauer spectroscopy without radioactive sources providing access to smaller samples and enabling shorter integration times (nuclear forward scattering) with unprecedented signal-to-noise ratio. In contrast to a Mössbauer source, synchrotron and X-ray free-electron laser radiation is emitted in short intense pulses which have an energy bandwidth in the eV range. This leads to the simultaneous excitation of the accessible nuclear levels of the nuclei in the sample. Accordingly, the nuclear resonant scattered radiation of the different energy levels interferes with each other, leading to a temporal beating pattern in the life-time of the excited states.

A key challenge for the detector systems in recording these time spectra is the handling of the large number of nonresonant, prompt photons. There are various techniques based on meV-monochromatization or temporal discrimination (Faigel *et al.*, 1987 ▸; Kishimoto, 1992 ▸; Toellner, 2000 ▸; Röhlsberger *et al.*, 2001 ▸; Toellner *et al.*, 2011 ▸), used individually or in combination, to discriminate the resonantly scattered photons from the energetically broadband synchrotron beam. One of these techniques is the polarization filtering method (Toellner *et al.*, 1995 ▸; Siddons *et al.*, 1995 ▸). This method uses the splitting of the energy levels of the atomic nucleus when an external magnetic field is applied, *i.e.* the nuclear Zeeman effect. A σ to π change in polarization of the resonantly scattered photons occurs for transitions with an angular momentum change of Δ*I* = ±1. Since no polarization change occurs for the non-resonantly scattered photons, it is possible to filter out the resonantly scattered photons with a linear polarization analyzer in crossed setting.

Due to the very strong rejection of the nonresonant ‘prompt’ radiation, one obtains access to the early times after excitation, which are important, *e.g.* for the reconstruction of magnetic spin structures from the nuclear forward scattering time spectra. To the best of our knowledge, Mössbauer spectroscopy combined with high-precision X-ray polarimetry has so far been restricted in practice to ^57^Fe (Siddons *et al.*, 1995 ▸; Toellner *et al.*, 1995 ▸; Röhlsberger *et al.*, 1997 ▸; Siddons *et al.*, 1999 ▸; Alp *et al.*, 2000 ▸; Heeg *et al.*, 2013 ▸; Heeg *et al.*, 2015 ▸; Marx-Glowna *et al.*, 2021 ▸), the most frequently used Mössbauer isotope. Polarization control for ^57^Fe Mössbauer radiation has also been demonstrated with a diamond phase plate (Mitsui *et al.*, 2015 ▸), which differs from the crossed high-purity polarizer technique discussed here. Extending high-purity polarimetry into the higher energy range above 14.4 keV would open the door to additional Mössbauer isotopes such as ^151^Eu, ^149^Sm, ^119^Sn and ^161^Dy, and thereby substantially broaden the range of materials and phenomena accessible to synchrotron Mössbauer spectroscopy.

In this work we address this challenge in the energy range 15–30 keV. We develop and implement an asymmetric channel-cut Si polarimeter optimized for operation near the Brewster angle at 22.494 keV, and we show that polarization purities at the 10^−8^ level can be achieved under realistic synchrotron beamline conditions. Supported by dynamical diffraction calculations, our results indicate that synchrotron Mössbauer spectroscopy can be extended to nuclear transitions up to approximately 30 keV, enabling new applications in condensed-matter physics and precision tests of fundamental interactions.

## 2. High-purity X-ray polarimetry

Typically, X-ray polarimeters based on Bragg reflections by crystals close to 45° (Hart, 1978 ▸; Hrdý *et al.*, 1979 ▸), which is the Brewster angle for X-rays, are used for the polarization filter method. For diffraction at the Brewster angle, the polarization component parallel to the diffraction plane (π-component) is suppressed in accordance with the dynamical theory for X-rays, while the σ-component is reflected.

The degree of freedom for the energy variation of this method is the choice of the crystal material, the lattice planes therein and the diffraction order *n*. The wavelength λ is related to the lattice plane spacing *d* of the crystal via the Bragg equation, 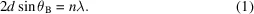
The best polarization suppression is achieved at the Brewster angle. However, for applications, like nuclear resonance scattering, the energy is fixed, so that one has to look for Bragg reflections from standard single crystals (silicon, germanium, diamond) that come as close as possible to the X-ray Brewster angle of 45°.

**Figure 1:**
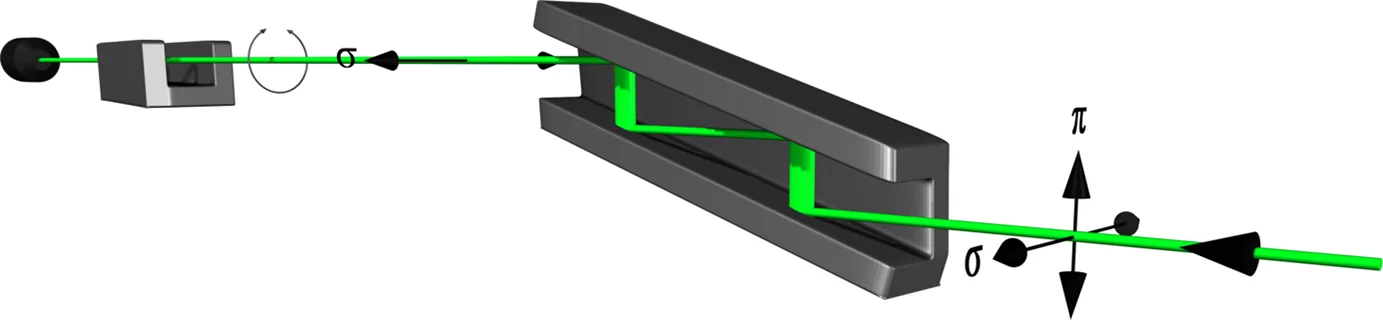
Schematic setup of a high-precision X-ray polarimeter. The radiation coming from the right is polarized by the first crystal. The second crystal in the extinction position almost completely suppresses the radiation.

To achieve a high polarization purity even in cases of deviations of the Bragg angle from the ideal 45°, one designs channel-cut crystals with more than two consecutive reflections as linear polarizers (Hart, 1978 ▸; Hart & Rodrigues, 1979 ▸), see Fig. 1 ▸.

The degree of polarization purity is defined as the ratio of the transmission of the π-component to the transmission of the σ-component (Alp *et al.*, 2000 ▸), 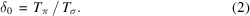
The transmission is a function of the brilliance of the X-ray source and the reflectivity of the crystal around the Bragg reflection. On the one hand, the degree of polarization purity is determined by the divergence, energy and energy bandwidth of the source; on the other hand, the parameters of the polarimeter crystals, that influence the reflectivity (crystal material, crystal orientation, number of crystal reflections), play a crucial role.

Undulators at third-generation X-ray sources, like PETRA III in Hamburg, Germany, can deliver a natural linear polarization purity of δ_0_ ≃ 10^−4^ (Marx *et al.*, 2014 ▸). Polarization purity levels of ∼10^−7^ are possible with the very long undulators at X-ray free-electron lasers (Geloni *et al.*, 2015 ▸). For many experimental applications, the natural degree of polarization of synchrotrons and X-ray free-electron lasers is sufficient so that only a single analyzer crystal is required for a reasonable polarization analysis. For high-precision X-ray polarimetry, as also required for Mössbauer spectroscopy, a high-purity polarizer crystal is needed in addition to the analyzer crystal to achieve degrees of polarization purity equal to or better than 10^−8^.

Fig. 1 ▸ shows the typical setup of an X-ray polarimeter. The incident unpolarized or partially polarized beam is polarized by the first channel-cut crystal. The second channel-cut crystal serves as an analyzer. The analyzer can be rotated around the beam incident from the polarizer to measure polarization rotations or to vary the suppression of the π-component. In the energy range up to 13 keV, polarization purity levels of 1.4 × 10^−11^ could be achieved with such precision polarimeters (Marx-Glowna *et al.*, 2022 ▸).

### 2.1. Limitations at higher energies

High-purity X-ray polarimeters, which are based on diffraction in perfect crystals, are subject to limitations with regard to their performance at higher X-ray energies. In the following, the influence of the full width at half-maximum (FWHM) of the reflection curve and multiple-wave diffraction is considered. We will focus in the following discussion on the Bragg geometry in the energy range between 15 keV and 30 keV.

#### 2.1.1. FWHM of the rocking curve

The angular FWHM of a perfect crystal reflection curve Δθ depends on the wavelength λ, the structure factor *F*_H_, the volume of the unit cell *V*, the polarization factor *C* and the Bragg angle θ_B_,
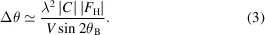
For polarimetry, the Bragg angle is fixed and, besides the obvious quadratic wavelength proportionality, the energy dependence is also contained in the structure factor. The structure factor is the sum of the complex atomic form factors over the unit cell, weighted by their respective phase factors. The structure factor is typically dominated by its real part. The contribution of the imaginary part to the width is usually negligible.

A narrow rocking curve width implies a highly selective angular and spectral acceptance of the device. The experimentally achievable efficiency therefore depends not only on the intrinsic device characteristics but also on the overlap between the device acceptance and the photon phase space provided by the radiation source and upstream beamline optics. Polarimetry at high energies inherently requires radiation sources with high brilliance and low beam divergence, as the intrinsically narrow rocking curve widths impose stringent constraints on the accepted photon phase space.

The narrow rocking curve width at higher photon energies is also challenging for the adjustment of a precision X-ray polarimeter. On the one hand, a high spectral photon flux and, on the other, a mechanically and temperature-stable setup with precision goniometers are necessary. Standard one-circle goniometers with gearboxes achieve a full step resolution of 0.9′′, which can easily be reduced to 0.1′′ with microstepping. The angular resolution can be improved by a sine drive equipped with piezo actuator and encoder system on top of the goniometer.

**Figure 2:**
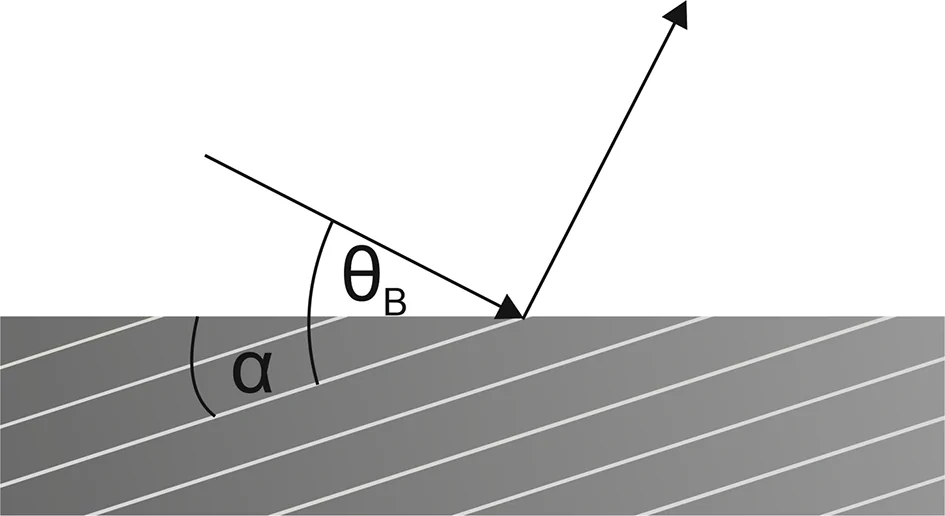
Asymmetrically cut crystal. The asymmetry angle α is the angle between the crystal surface and the diffracting lattice planes. For the grazing incidence case shown in the image, the angle is negative by definition.

The difficulty of narrow rocking curve widths in the higher energy range lies not only in positioning the rocking curve maximum but also in the stable mounting of the polarimeter crystals in the hundredths of an arcsecond range. In order to increase the angular acceptance of the crystal reflections, it is possible to cut them asymmetrically, *i.e.* the reflecting lattice planes are not parallel but tilted relative to the surface (see Fig. 2 ▸). The asymmetry angle α describes the tilt angle of the lattice planes to the surface. Angle α is negative if the angle of incidence to the crystal surface is smaller than the Bragg angle and correspondingly positive if the angle of incidence is greater than the Bragg angle. As a result of the asymmetric reflection, the rocking curve width on the input side of the crystal Δθ_−_ changes to 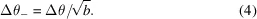
The asymmetry parameter *b* contained therein quantifies the asymmetry,
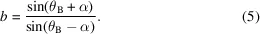
The smaller the asymmetry parameter becomes, the larger the angular acceptance on the input-side of the reflection becomes.

This is accompanied by a widening of the beam cross-section on the output side *S*_+_,
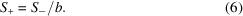
Table 1 ▸ shows the influence of the asymmetry angle on the rocking curve width and the degree of polarization purity for different numbers of crystal reflections *i* using Si (5 7 11) for ^149^Sm as an example.

A smaller asymmetry angle increases the FWHM of the rocking curve and leads to a slight improvement in the degree of polarization purity. Simultaneously, the footprint of the beam in the crystal increases and the peak reflectivity decreases. However, the asymmetry angle in channel-cut crystals cannot be chosen arbitrarily large, as the penetration depth of the X-rays into the crystal is reduced with smaller asymmetry angles and is therefore more sensitive to defects in the surface of the crystals. The footprint of the beam is also a limiting factor for the choice of asymmetry angle for materials such as diamond. The technically feasible crystal size for diamonds of high quality is in the 12 mm range (Freund *et al.*, 2001 ▸).

In addition to the FWHM values of the rocking curves presented in Table 1 ▸, the effect of the rocking-curve width on the efficiency is of particular importance. For two relevant scenarios the corresponding efficiencies were calculated, assuming an upstream Si (111) monochromator and an incident beam divergence Δθ_source_ of 2 µrad, which can be achieved at modern synchrotron beamlines using collimating optics (Sergueev *et al.*, 2025 ▸).

First the efficiency was calculated for the full spectral bandwidth provided by the Si (111) monochromator,
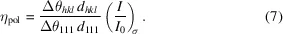
The reflecting plane spacing is denoted as *d*_*hkl*_. This efficiency is particularly relevant for the alignment and optimization of the instrument. If the transmitted intensity is too low, achieving and experimentally verifying high degrees of polarization purities becomes increasingly challenging due to insufficient photon statistics.

Second, the efficiency was calculated for the narrow energy bandwidth of the nuclear resonance representative of the intended application. In the case that the divergence of the source Δθ_source_ is smaller than the FWHM of the reflection of the Si (111) monochromator, the efficiency for the bandwidth of the resonance can be determined as follows,
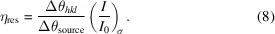
As evident from the values summarized in Table 1 ▸, the efficiency within the narrow energy bandwidth of the nuclear resonance exceeds the efficiency corresponding to the full monochromator bandwidth by approximately two orders of magnitude. The reduction in efficiency by roughly one order of magnitude for the analyzer crystal alone, and by about two orders of magnitude for the combined polarizer–analyzer configuration, places stringent requirements on source brilliance and nuclear resonant absorption cross sections, thereby favoring the use of high-brilliance radiation sources for Mössbauer spectroscopy.

#### 2.1.2. X-ray multiple-wave diffraction

The current range of application of precision X-ray polarimetry is in the energy range between 6.4 keV and 14.412 keV (Marx *et al.*, 2013 ▸; Heeg *et al.*, 2015 ▸; Schmitt *et al.*, 2021 ▸; Yu *et al.*, 2023 ▸). One of the contributing factors is that the number of possible Bragg reflections increases with increasing photon energy in a crystal, leading to a higher probability of satisfying additional Bragg conditions and thus to stronger multiple-wave diffraction effects. Since the rocking curves have very broad tails, the influence of multiple-wave diffraction on the degree of polarization purity increases and in general the polarization purity will be worse than the theoretical limit in the two-beam case. Multiple-wave diffraction, also referred to as *n*-beam diffraction, takes into account the simultaneous diffraction of multiple beams in an extension of the widely used two-beam case (Bragg condition) of the dynamical theory of X-ray diffraction.

**Figure 3:**
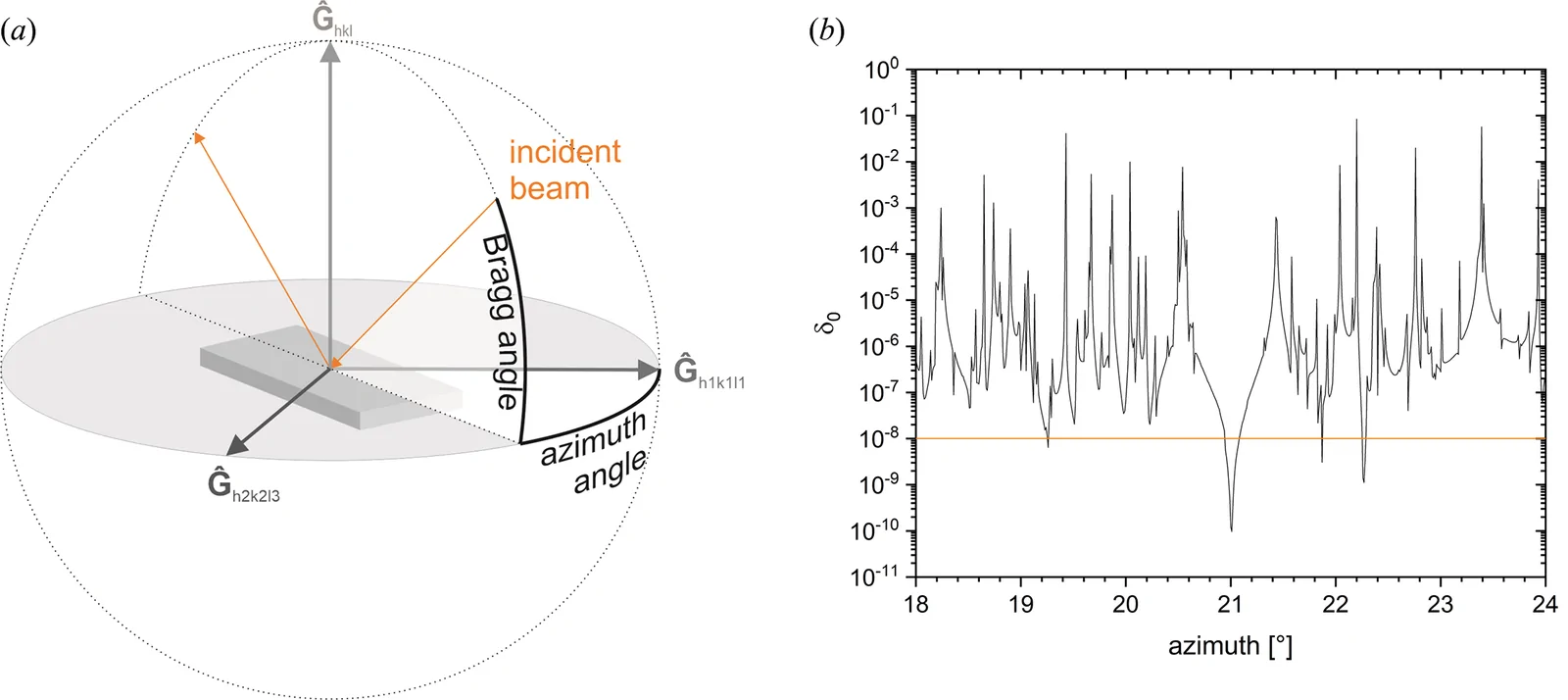
(*a*) Illustration of the azimuthal crystal orientation. (*b*) Variation of the degree of polarization purity as a function of the azimuthal orientation of the crystal for the 5 7 11 Bragg reflection of silicon at *E* = 22.494 keV. The simulation was carried out using the multiple-wave diffraction algorithm (Schulze, 2018 ▸). The azimuthal angle is measured relative to the crystallographic 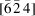 direction. The orange line indicates the polarization suppression of 10^−8^ (see Table 3 ▸) required for the polarimeter when used in Mössbauer spectroscopy.

In order to minimize the influence of multiple-wave diffraction, one can use the remaining degree of freedom, namely to rotate the polarimeter crystals azimuthally [see Fig. 3 ▸(*a*)], *i.e.* around an axis that stands perpendicular to the diffracting lattice planes (Renninger, 1937 ▸). Fig. 3 ▸(*b*) illustrates the variation of the degree of polarization purity as a function of the azimuthal orientation using samarium as an example.

For the energy of 22.494 keV as chosen here, the best crystal orientation is at an azimuth angle of 21°. This is where the ‘funnel’ in which the required purity is achieved is the largest such that the required orientation accuracy for the polarimeter crystals is the lowest.

The higher the photon energy, the more precisely the azimuthal orientation of the crystals has to be adjusted to achieve high degrees of polarization purity. Table 2 ▸ displays the achievable degree of polarization purity for selected nuclear resonances for the specified crystal orientation. In addition, the required accuracy of the azimuthal adjustment of the crystals for a degree of polarization purity of δ_0_ = 10^−8^ is indicated. Although the orientation requirements for the crystals increase, very good degrees of polarization purity can also be achieved in the higher energy ranges.

Another possibility to reduce the influence of multiple-wave diffraction is to choose a crystal material with a low atomic number, since the influence of multiple-wave diffraction scales with the fourth power of the atomic number of the element the channel-cut is made of (Tischler & Batterman, 1986 ▸). Diamond with atomic number *Z* = 6 is therefore a very suitable polarimeter crystal material.

### 2.2. Application: Mössbauer spectroscopy at photon energies above 22 keV

As an application example, high-purity polarimetry at high photon energies for Mössbauer spectroscopy will be considered in the following section.

Numerous Mössbauer isotopes are in the energy range 15–30 keV. Table 3 ▸ shows a number of selected Mössbauer isotopes with corresponding resonance energy. To estimate the required polarization suppression for the polarization filtering method, the ratio between the nuclear resonance energy bandwidth Γ_0_ and the energy bandwidth of a Si(111) monochromator is considered in the third column. These monochromators are the standard at the third- and fourth-generation sources. The narrower the energy bandwidth of the resonance, the higher the requirements for the polarization purity of the polarimeter, as this increases the proportion of prompt photons to be filtered out. Secondly the ratio of the nuclear resonance absorption cross section to the total electronic absorption cross section is shown in the fourth column. The lower the value, the better the polarization suppression should be to achieve a good signal-to-noise ratio in the measurements. For most resonances, a polarization purity of the polarimeter of 10^−8^ is sufficient to keep detector afterpulsing under control during Mössbauer spectroscopy measurements.

The following columns in the table list the reflections closest to the Brewster angle for the chosen Mössbauer isotope with transition energy *E*_γ_. The table indicates that many nuclear resonances can indeed be addressed with one of the standard single crystals. Germanium is not included in Table 3 ▸. Above approximately 14 keV, the extinction length in germanium becomes comparable with the absorption length, reducing the peak reflectivity of the rocking curves. While certain reflections may still be suitable for two-reflection polarizers, the reduced reflectivity makes germanium less attractive for multi-reflection channel-cut designs due to the cumulative reflectivity losses.

Furthermore, Table 3 ▸ lists the FWHM values Δθ_σ_ for the σ-component of the given reflection. The values show that, for the energy range above 20 keV, the FWHM of the rocking curves is just a few 0.01′′. The considerably narrower acceptance of the polarimeter crystal results in a reduced phase-space overlap with the beam transmitted by the Si(111) monochromator, leading to a lower transmitted photon flux according to equation (7).

The last column in Table 3 ▸ shows the polarization purity δ_0_ that can be achieved with *i* = 2 symmetric reflections. It can be seen that the polarization purity deteriorates the further the Bragg angle deviates from the Brewster angle. This can be counteracted by increasing the number of successive Bragg reflections in channel-cut crystals (Hart, 1978 ▸). Unfortunately, this goes with a reduction of the peak reflectivity and requires larger single crystals or greater technical effort like realizing an artificial channel-cut crystal (quasi-channel-cut) (Bernhardt *et al.*, 2020 ▸). For strongly asymmetrical reflections, only two reflections are technically practicable, as otherwise the beam path in the channel-cut crystal and the crystal size become too large.

## 3. Demonstration experiment

To demonstrate the feasibility of high-purity X-ray polarimetry at high photon energies, an X-ray polarimeter was developed for the samarium nuclear resonance at 22.494 keV and tested at beamline P24 at PETRA III (DESY, Hamburg, Germany). The beamline provides an energy bandwidth of 2.9 eV at 22.494 keV employing a water-cooled Si (111) monochromator with 50 µm copper absorber. The photon energy transmitted by the monochromator was measured using the Bond method (Bond, 1960 ▸). The beam size was defined to 1 mm × 1 mm by a slit behind the monochromator.

Two four-reflection silicon (5 7 11) channel-cut crystals with an asymmetry angle of α_1_ = α_3_ = −28° and α_2_ = α_4_ = 28° were used for the polarimeter. The azimuthal orientation of the polarimeter crystals was chosen as 21.0° with respect to 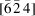 and 64.9° with respect to 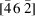 and was aligned optically via back-reflection of a laser beam from the outer surfaces of the crystal. The Bragg angle alignment of the polarizer crystal was performed using a conventional full-circle goniometer with a full-step resolution of 0.9′′. To monitor the positioning of the polarizer during the measurements, an ionization chamber was installed between the two polarizer channel-cut crystals. In addition to the conventional goniometer, a piezo-driven sine drive (P 401, HUBER Diffraktionstechnik GmbH & Co. KG) with an angular resolution of 0.02′′ was used to align the analyzer crystal on the Bragg reflection.

**Figure 4:**
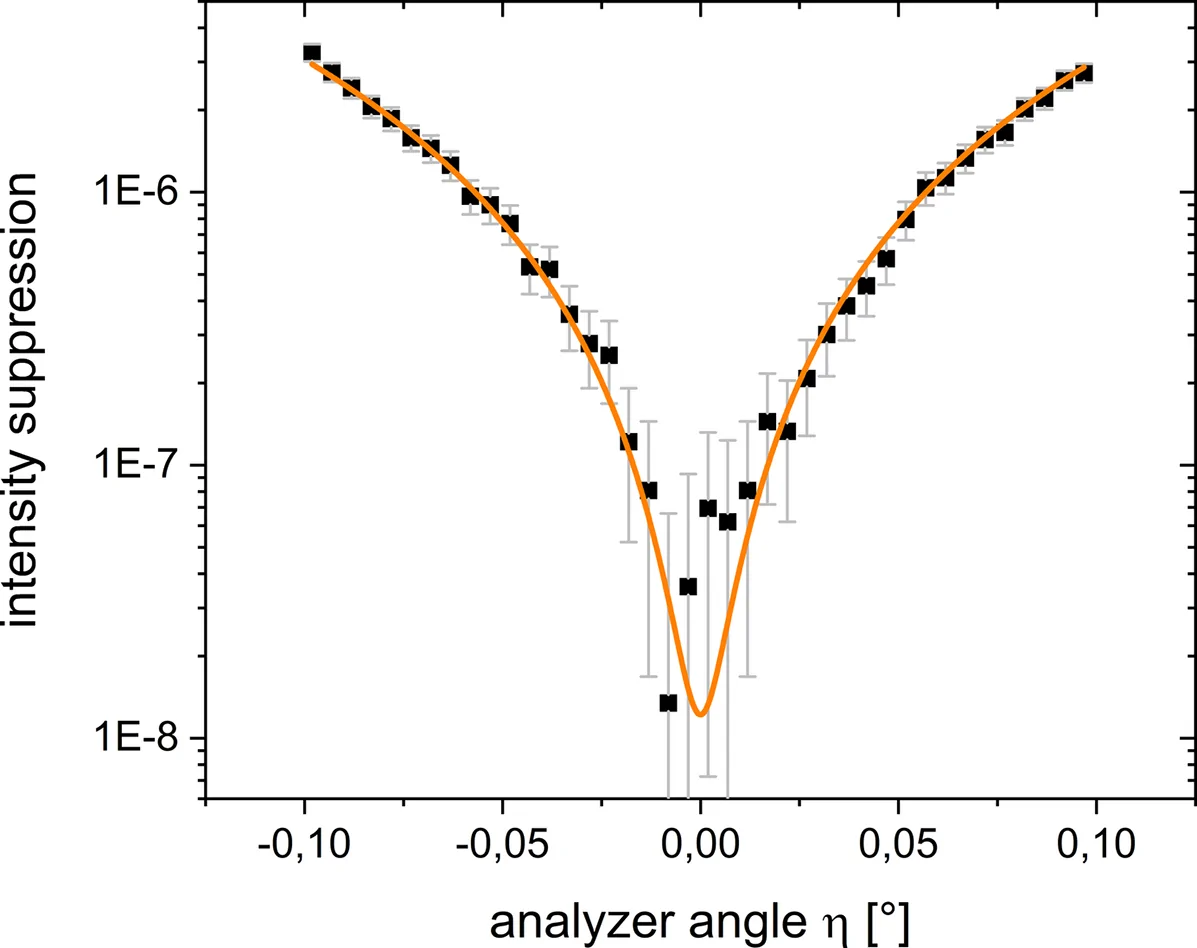
Measurement of the polarization purity for four-reflection Si (5 7 11) channel-cuts at a photon energy of 22.494 keV. The orange solid line corresponds to a fit with second-order polynomial. The vertical error bars represent the statistical uncertainties of the data points and the background measurement, assuming Poisson statistics with a 95% confidence level.

The analyzer crystal can be rotated to the extinction position (η circle) using a third goniometer which also has a full step resolution of 0.9′′. During the measurement, the synchrotron was operating in the multibunch filling mode at a storage ring current of 100 mA. The detector used was an AdvaPIX TPX3 with a CdTe sensor of 1 mm thickness. Fig. 4 ▸ shows the result of the polarization purity measurement near the extinction position (η = 0°) of the polarimeter. The measurement duration per data point was 100 s. The count rate at η = 0° amounts to 0.43 counts s^−1^. The measurement duration for the required subtraction of the background in the extinction position was 1000 s. The vertical beam divergence at P24 was approximately 136 µrad (∼28′′), far larger than both the angular acceptance of the channel-cut crystals (∼0.06′′) and the 2 µrad divergence assumed for the efficiencies listed in Table 1 ▸; the beamline used here therefore transmitted a substantially lower photon flux than an optimized high-brilliance beamline would. This reduced flux accounts for the low count rate of 0.43 counts s^−1^. A degree of polarization purity of (1.2 ± 0.5) × 10^−8^ was achieved, which is sufficient for the intended synchrotron Mössbauer spectroscopy (third column of Table 3 ▸). Further optimization of the crystal azimuthal orientation, for example via beam divergence optimization and background suppression, could in principle improve the result toward the theoretical value.

## 4. Conclusion

This work has shown that high-purity X-ray polarimetry, previously demonstrated only up to photon energies of about 14.4 keV, can in principle be extended to at least 30 keV. In this energy regime two experimental challenges become particularly critical: the extremely narrow rocking-curve widths and the increased influence of multiple-wave diffraction. Here these limitations were addressed using established approaches from dynamical-diffraction theory—asymmetric reflections combined with piezo-driven goniometers for precise angular control, and optimized azimuthal crystal orientations to minimize multiple-wave effects. On this basis, a proof-of-principle polarimeter for 22.494 keV was realized at PETRA III, reaching a polarization purity of (1.2 ± 0.5) × 10^−8^ even without azimuthal fine tuning—already sufficient for synchrotron Mössbauer spectroscopy. A nuclear-resonant measurement on ^149^Sm, which requires a dedicated high-brilliance beamline, is the natural next step and was beyond the scope of the present optics development.

High-purity X-ray polarimetry at high energies offers promising applications in Mössbauer spectroscopy. In particular, the nuclear resonance of ^149^Sm enables precise studies of magnetic phenomena at phase transitions. Samarium-based pyrochlore structures are especially relevant, as geometric frustration gives rise to unconventional magnetic behavior (Singh *et al.*, 2008 ▸; Sain & Upadhyay, 2024 ▸). More generally, our results pave the way for systematic synchrotron Mössbauer experiments on high-energy isotopes and for precision X-ray polarimetry applications such as tests of QED vacuum birefringence (Karbstein & Sundqvist, 2016 ▸).

## Figures and Tables

**Table 1:** Influence of the asymmetry parameter α on the FWHM Δθ and maximum peak intensity *I*/*I*_0_ of the 5 7 11 Bragg reflection of silicon at a photon energy of 22.494 keV 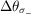
 denotes the FWHM of the rocking curve of the σ-polarization component on the input side. The case *i* = 1 is shown solely to illustrate the difference compared with multiple reflections. Polarizer and analyzer are typically cut identically with an even number of reflections. Furthermore, the footprint of the beam of 1 mm diameter on the crystal surface and the achievable degree of polarization purity is indicated. The values marked with an asterisk (*) are limited by multiple-wave diffraction. Efficiencies are given for the Si(111)-monochromated beam (η_pol_) and for the application-specific energy bandwidth, assuming a beam divergence of 2 µrad (η_res_). The efficiencies η_pol_ and η_res_ are given per channel-cut crystal; for a polarimeter the corresponding values for polarizer and analyzer combine multiplicatively.

α (°)	Footprint (mm)	*i*	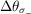 (′′)	(*I*/*I*_0_)_σ_	δ_0_	η_pol_	η_res_
0	1.4	1	0.04	0.88	1.1 × 10^−4^	1.8 × 10^−3^	8.5 × 10^−2^
		2	0.04	0.78	1.0 × 10^−7^	1.6 × 10^−3^	7.5 × 10^−2^
		4	0.03	0.61	4.0 × 10^−10^*	9.5 × 10^−4^	4.4 × 10^−2^
−28	3.4	1	0.07	0.86	9.7 × 10^−5^	3.1 × 10^−3^	1.5 × 10^−1^
		2	0.06	0.75	6.8 × 10^−8^	2.3 × 10^−3^	1.1 × 10^−1^
		4	0.06	0.56	4.0 × 10^−10^*	1.8 × 10^−3^	8.1 × 10^−2^
−41	13.9	1	0.14	0.77	6.3 × 10^−5^	5.6 × 10^−3^	2.6 × 10^−1^
		2	0.13	0.60	1.8 × 10^−8^	4.1 × 10^−3^	1.9 × 10^−1^

**Table 2:** Optimal azimuthal orientation for polarimeter crystals for selected Mössbauer isotopes The crystal orientation can be specified by one or two non-collinear reference directions perpendicular to the reciprocal lattice vector. The achievable purity δ_0_ and the orientation accuracy β of the polarimeter crystals required for δ_0_ = 10^−8^ are specified. The values marked with an asterisk (*) are limited by the divergence and depend on the X-ray source used.

Isotope	*E*_γ_ (keV)	Reflection		δ_0_	Orientation		β
^181^Ta	6.214	Ge	4 0 0	<1 × 10^−11^*	0°	[0 1 1]	6.3°
^45^Sc	12.390	Si	5 5 3	4 × 10^−11^	0°	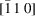	1.9°
^149^Sm	22.494	Si	5 7 11	4 × 10^−10^	21° / 64.9°	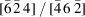	0.1°
^40^K	29.834	C	11 5 1	2 × 10^−9^	44.79° / 22.1°	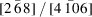	0.06°

**Table 3:** Overview of selected Mössbauer isotopes (Röhlsberger, 2004 ▸) with corresponding resonance energy *E*_γ_ To estimate the necessary degree of polarization purity of the polarimeter, the third and fourth columns show the ratio of the energy bandwidth of the nuclear resonance to the energy bandwidth of a silicon monochromator as well as the ratio of the nuclear resonance absorption cross section σ_0_ to the total electronic absorption cross section σ_ph_. The crystal reflections that can be used for the polarization filter method are listed, together with the associated Bragg angles θ_B_ and FWHM of the rocking curve Δθ. The purity calculations δ_0_ for two symmetric crystal reflections (*i* = 2) are shown solely to demonstrate the dependence on the deviation from the Brewster angle.

				Silicon				Diamond			
Isotope	*E*_γ_ (keV)	Γ_0_/Δ*E*_Si(111)_	σ_0_/σ_ph_	Reflection	θ_B_ (°)	Δθ_σ_ (′′)	δ_0_ (*i* = 2)	Reflection	θ_B_ (°)	Δθ_σ_ (′′)	δ_0_ (*i* = 2)
^57^Fe	14.4125	3 × 10^−9^	428.58	8 4 0	45.103	0.39	1.6 × 10^−7^	5 3 1	45.512	0.48	5.6 × 10^−3^
^151^Eu	21.532	2 × 10^−8^	29.05	13 3 1	45.174	0.05	3.3 × 10^−7^	7 5 1	44.347	0.15	3.7 × 10^−3^
				11 7 3	45.174	0.05	3.3 × 10^−7^				
				9 7 7	45.174	0.05	3.3 × 10^−7^				
^149^Sm	22.494	2 × 10^−8^	17.29	5 7 11	45.122	0.04	7.6 × 10^−8^	9 1 1	44.74	0.13	9.0 × 10^−5^
				13 5 1	45.122	0.04	7.6 × 10^−8^	7 5 3	44.74	0.13	9.0 × 10^−5^
^119^Sn	23.871	8 × 10^−9^	562.59	13 7 1	45.042	0.03	1.0 × 10^−9^	8 4 4	45.51	0.15	1.8 × 10^−3^
				13 5 5	45.042	0.03	1.0 × 10^−9^				
^161^Dy	25.65135	5 × 10^−9^	176.22	15 5 1	44.829	0.02	2.3 × 10^−7^	9 5 1	44.494	0.08	4.8 × 10^−4^
				11 11 3	44.829	0.02	2.3 × 10^−7^	7 7 3	44.491	0.08	4.8 × 10^−4^
				11 9 7	44.829	0.02	2.3 × 10^−7^				
				13 9 1	44.829	0.02	2.3 × 10^−7^				
^201^Hg	26.269	2 × 10^−7^	1.80	16 2 2	44.912	0.03	3.8 × 10^−8^	9 5 3	45.193	0.07	8.3 × 10^−6^
				14 8 2	44.912	0.03	3.8 × 10^−8^				
				10 10 8	44.912	0.03	3.8 × 10^−8^				
^129^I	27.78	5 × 10^−9^	194.18	16 6 2	44.985	0.02	2.8 × 10^−11^	8 8 0	45.056	0.09	1.1 × 10^−7^
				17 3 1	45.275	0.01	1.1 × 10^−6^				
^40^K	29.834	3 × 10^−8^	1336.74	18 4 2	45.204	0.01	7.2 × 10^−7^	11 5 1	44.934	0.05	1.2 × 10^−11^
				17 7 1	44.784	0.01	3.9 × 10^−7^				
				17 5 5	44.784	0.01	3.9 × 10^−7^				

## Data Availability

The data reported in the article are available upon request to the corresponding author.
